# Safety profile of the intravenous administration of brain-targeted stable nucleic acid lipid particles

**DOI:** 10.1016/j.dib.2016.01.017

**Published:** 2016-01-20

**Authors:** Mariana Conceição, Liliana Mendonça, Clévio Nóbrega, Célia Gomes, Pedro Costa, Hirokazu Hirai, João Nuno Moreira, Maria C. Lima, N. Manjunath, Luís Pereira de Almeida

**Affiliations:** a CNC – Center for Neuroscience and Cell Biology, University of Coimbra, Rua Larga, 3004-504 Coimbra, Portugal; b Faculty of Pharmacy, University of Coimbra, 3000-548 Coimbra, Portugal; c IBILI – Institute of Biomedical Research in Light and Image, Faculty of Medicine, University of Coimbra, 3000-354 Coimbra, Portugal; d Graduate School of Medicine, Gunma University, Maebashi, Gunma 371-8511, Japan; e Center of Excellence in Infectious Diseases, Paul L. Foster School of Medicine, Texas Tech University Health Sciences Center, El Paso, TX 79905, USA

## Abstract

In a clinical setting, where multiple administrations of the therapeutic agent are usually required to improve the therapeutic outcome, it is crucial to assess the immunogenicity of the administered nanoparticles. In this data work, we investigated the safety profile of the repeated intravenous administration of brain-targeted stable nucleic acid lipid particles (RVG-9r-targeted SNALPs). To evaluate local activation of the immune system, we performed analysis of mouse tissue homogenates and sections from cerebellum. To investigate peripheral activation of the immune system, we used serum of mice that were intravenously injected with RVG-9r-targeted SNALPs. These data are related and were discussed in the accompanying research article entitled “Intravenous administration of brain-targeted stable nucleic acid lipid particles alleviates Machado–Joseph disease neurological phenotype” (Conceição et al., in press) [1].

## Specifications table

**Table:** 

Subject area	*Biology/Neurosciences*
More specific subject area	*Immunology*
Type of data	*Text, figures with microscopy images and graphs, tables*
How data was acquired	*Microscope (Zeiss PALM Axiovert 200M), Real-time PCR system (Step One Plus Real-Time PCR System, Applied Biosystems), Spectrophotometer (Spectramax Plus 384, Molecular Devices). Results were analyzed using GraphPad Prism software*
Data format	*Analyzed*
Experimental factors	*Mice were intravenously injected with brain-targeted stable nucleic acid lipid particles*
Experimental features	*Mouse serum, mouse brain sections and mouse cerebellar homogenates were analyzed to evaluate the immune response*
Data source location	*Center for Neurosciences and Cell Biology, University of Coimbra, Coimbra, Portugal*
Data accessibility	*Data is supplied in this article*

## Value of the data

• Provide an insight of the tolerability of brain-targeted SNALPs.
• Evaluate whether intravenous administration of RVG-9r-targeted SNALPs would contribute to inflammation, which could preclude repeated intravenous administration.
• Compare to other data that show the immune response upon administration of siRNA delivery systems.
• Constitute a support for the future development of non-viral vectors as therapeutic vehicles.

## 1 Data

**Fig. 1:**
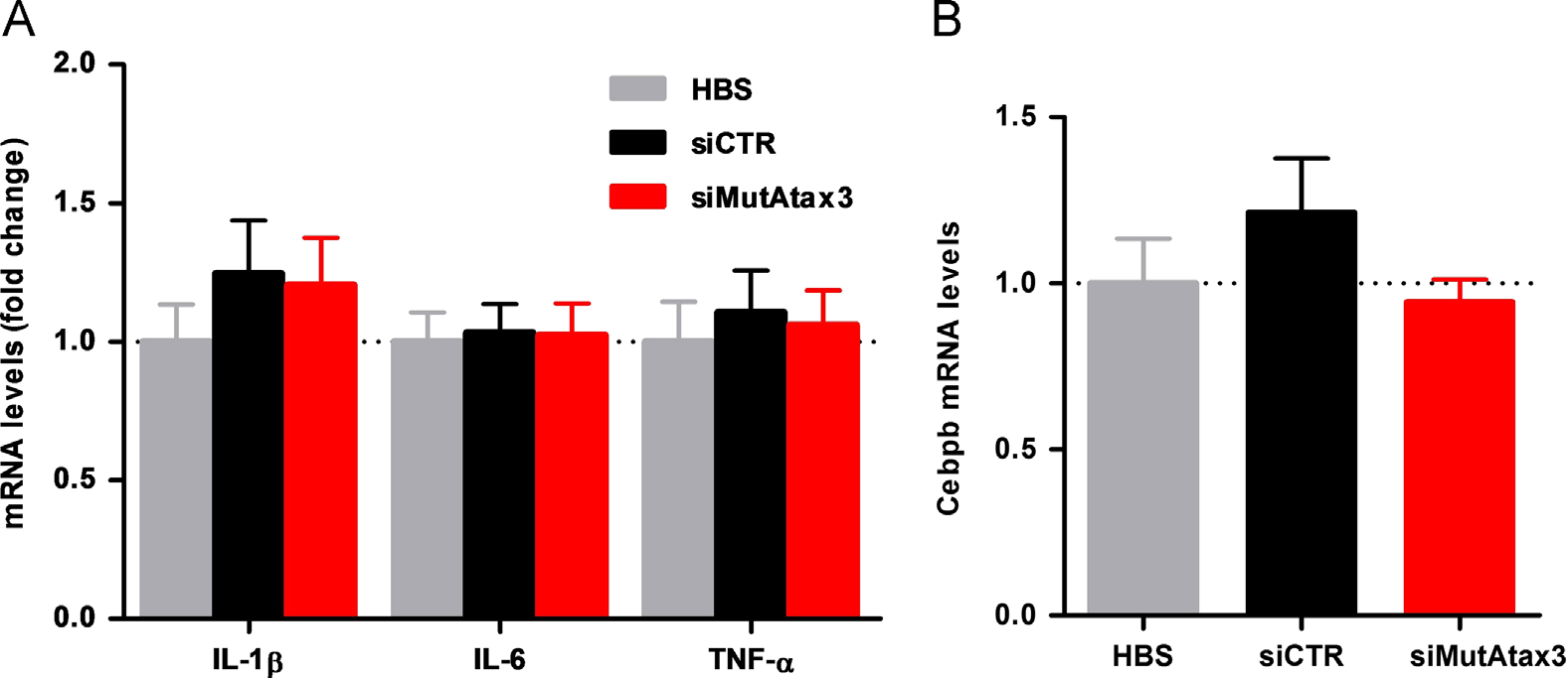
Intravenous administration of RVG-9r-targeted SNALPs does not stimulate the production of pro-inflammatory cytokines neither the activation of microglia. (A) mRNA relative levels of pro-inflammatory mediators (IL-1β, IL-6 and TNF-α) in the cerebellum of MJD transgenic mice injected with RVG-9r-targeted SNALPs (black – siCTR, red – siMutAtax3) relatively to animals injected with HBS (gray). Results were normalized using HPRT housekeeping gene. (B) mRNA relative levels of a microglia-related gene – Cebpb – in the cerebellum of MJD transgenic mice. Values are presented as mean±SEM of at least 4 independent experiments. One-way ANOVA analysis of variance combined with Bonferroni’s post-test was used for multiple comparisons (n.s. *p*>0.05).

Nucleic acids are potential activators of the immune system and can stimulate the production of high levels of inflammatory cytokines such as tumor necrosis factor-alpha (TNF-α), interleukin-6 (IL-6) and interferons [2], [3]. Another potent inflammatory cytokine that is highly produced in the central nervous system as a response to damage, stress or disease is interleukin-1beta (IL-1β). To investigate whether intravenous administration of RVG-9r-targeted SNALPs would contribute to inflammation, which could preclude repeated administration, we first evaluated the mRNA levels of pro-inflammatory cytokines (IL-1β, IL-6 and TNF-α) and a microglia-related gene (Cebpb) in the cerebella of these animals. No significant differences were detected in the mRNA levels of these mediators between HBS-injected mice or mice injected with RVG-9r-targeted SNALPs (Fig. 1A and B).

**Fig. 2:**
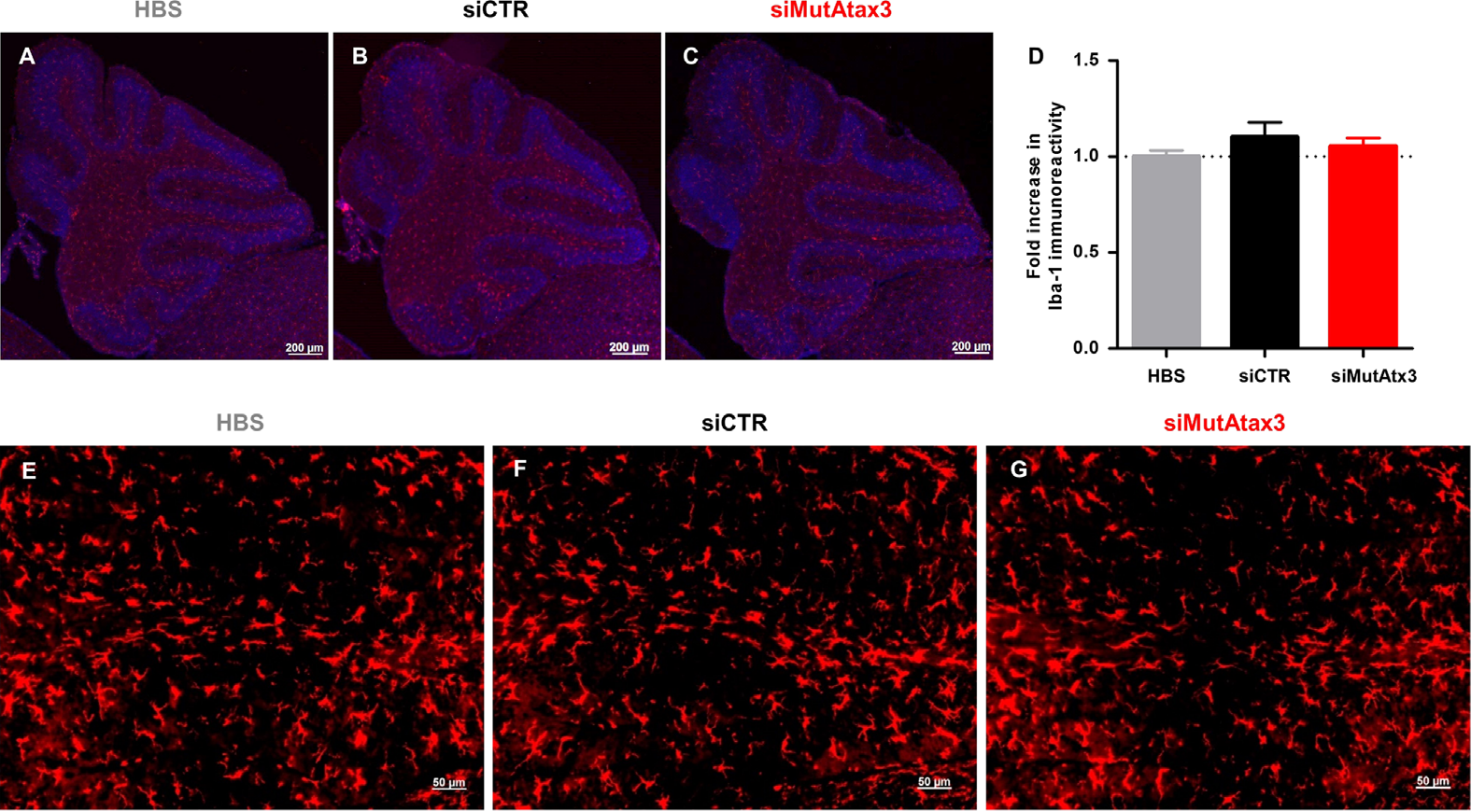
Repeated intravenous administration of RVG-9r-targeted SNALPs does not elicit local microglial activation. 8 weeks after starting the treatment, mice were sacrificed and Iba-1 staining was evaluated by fluorescence immunohistochemistry. (A–C) Fluorescence microscopy images of microglia stained with Iba-1 antibody, with a 5× objective. (D) Quantification of the Iba-1 immunoreactivity. Values are presented as mean±SEM of *n*=5/6. One-way ANOVA analysis of variance combined with Bonferroni’s post-test was used to compare animals injected with HBS versus siMutAtax3 and siCTR (n.s. *p*>0.05). (E–G) Fluorescence microscopy images of microglia stained with Iba-1 antibody, with a 20× objective.

To evaluate whether repeated intravenous administration of RVG-9r-targeted SNALPs would activate microglia, we measured Iba-1 immunoreactivity in the cerebellum. When compared to HBS-injected animals, no significant increase in Iba-1 immunoreactivity was detected for the animals injected with RVG-9r-targeted SNALPs (Fig. 2).

Given that a previous study suggested that intravenous administration of cationic complexes incorporating siRNAs and RVG-9r strongly increased the levels of the IL-6 pro-inflammatory cytokine [4], we evaluated whether RVG-9r-targeted SNALPs would transiently increase IL-6 levels. Four hours after intravenous administration of RVG-9r-targeted SNALPs, we detected a significant increase in the levels of IL-6 (40±12.5 pg/mL, Table 1), albeit smaller than what was previously reported [4].

Lastly, we evaluated whether repeated intravenous administration of RVG-9r-targeted SNALPs (over a 2-months period) would increase the IL-6 levels. When compared to animals injected with a saline solution, repeated intravenous administration of RVG-9r-targeted SNALPs did not increase chronically the levels of IL-6 (Table 2).

## 2 Experimental design, materials and methods

### 2.1 Detection of the cerebellar mRNA levels of inflammatory mediators

5-weeks-old C57 BL/6 ataxin-3 [Q69] transgenic mice were intravenously injected on 3 consecutive days with 2.5 mg/kg of siRNA (siCTR or siMutAtax3) encapsulated in RVG-9r-targeted liposomes or HEPES-buffered saline solution (HBS). To analyze mutant ataxin-3 levels, mouse cerebella were harvested 48 h after the third injection. To detect cerebellum mRNA levels, total RNA was extracted using Qiazol solution (Qiagen) followed by purification of the RNA product using the Nucleospin RNA kit (Macherey-Nagel). cDNA synthesis was performed using the iSCRIPT cDNA synthesis kit (Bio-Rad). To determine the levels of IL-1β, IL-6 and TNF-α we used 2.5 µL of cDNA diluted 4 times (for Cebpb cDNA was diluted 10 times) and annealing temperature of 61 °C. qRT-PCR was performed using SsoAdvanced™ Universal SYBR® Green Supermix (Bio-Rad), in a Step One Plus Real-Time PCR System (Applied Biosystems). The StepOne Software generated automatically threshold cycle (Ct) values and the levels of the target gene were calculated using HPRT as housekeeping gene. The mRNA relative quantification was performed with the Pfaffl method relatively to control samples.

### 2.2 Fluorescence immunohistochemistry

The animals used for this study were the animals that were subjected to behavior assessment in our accompanying research article [1]. These animals were tail-vein injected with 2.5 mg/kg of siRNAs (each injection) encapsulated in RVG-9r-targeted liposomes and each mouse received a total of nine tail-vein injections during the time-course of the experiment.

To determine whether repeated intravenous administration of RVG-9r-targeted SNALPs elicit local microglial activation, brain tissue was prepared as we described in our accompanying paper [1]. Immunohistochemical procedure was performed as we previously reported [5] with very few alterations. Briefly, the protocol was initiated with 1 h of blocking and permeabilization in 0.3% Triton X-100 10% normal goat serum prepared in PBS at room temperature. Sections were then incubated overnight at 4 °C with the primary antibody rabbit polyclonal anti-Iba1 (1:1000, Wako) that was prepared in 2% normal goat serum 0.05% Triton X-100 (in PBS). Sections were washed three times with PBS and incubated with goat-anti-rabbit conjugated to Alexa Fluor 594 (1:250, Molecular Probes) prepared in 2% normal goat serum (Gibco) for 2 h at room temperature. Nuclei staining was performed with DAPI, sections were washed three times with PBS and then mounted on gelatinized slides. After being dried, slides were mounted in Mowiol reagent. Widefield fluorescence images were acquired with 5× and 20× objective on a Zeiss PALM Axiovert 200M imaging microscope.

Quantitative analysis of the Iba-1 immunoreactivity was performed by scanning 6 stained-sections per animal that were distanced 240 µm from each other. Cerebella were imaged using the MosaiX feature of the Axiovision software (Zeiss) using Zeiss PALM Axiovert 200M microscope with 5× objective. The immunoreactivity indexes were measured through optic density analysis for the entire cerebellum, using an image-analysis software (Image J software, NIH, USA).

### 2.3 Quantification of IL-6 serum levels by ELISA

For these studies we used mice that were subjected to one or several intravenous administrations of RVG-9r-targeted SNALPs. For mice that were intravenously injected with RVG-9r-targeted SNALPs only once, IL-6 serum levels were evaluated 4 h after injection. For the animals that were repeatedly intravenously injected over a 2-months period (animals that were subjected to behavior assessment), the levels of serum IL-6 were measured 5 days after the last injection.

Quantification of IL-6 serum levels was performed in duplicate, using 50 µL of serum and the Mouse IL-6 ELISA Kit from Merck Millipore according to the manufacturer’s protocol. The absorbance was read at 450 nm and 570 nm in a Spectramax Plus 384 spectrophotometer (Molecular Devices), and the absorbance at 570 nm was subtracted from the absorbance at 450 nm. As a positive control, one wild-type animal (with approximately 20 g) was intraperitoneally injected with 200 µg of lipopolysaccharides (LPS) from *Escherichia coli* O26:B6.

### 2.4 Statistical analysis

Data are presented as mean±SEM of at least 4 independent experiments. Student’s *t*-test or one-way ANOVA combined with Bonferroni’s post-test were used to determine statistically significant differences of the mean. Statistical differences are presented at probability levels of *p<0.05, **p<0.01 and ***p<0.001 and considered non-significant (n.s.) when *p*>0.05. Calculations were performed using GraphPad Prism version 5.00 for Windows.

## Figures and Tables

**Table 1:** Intravenous administration of RVG-9r-targeted SNALPs increase IL-6 serum levels. The levels of IL-6 for non-injected wild-type mice or mice intravenously injected with RVG-9r-targeted liposomes encapsulating siMutAtax3 (siMutAtax3) were measured by ELISA, 4 h after tail-vein injection. Values are presented as mean±SEM of *n*=4. Student’s *t*-test with Welch’s correction was used to compare non-injected animals to animals injected with RVG-9r-targeted SNALPs.

**Animal**	**Wild Type**	
**Non-injected**	**RVG-9r-Liposomes siMutAtax3**
**IL-6 levels (pg/mL)**	0	39.75±12.5*

* *p*<0.05.

**Table 2:** Repeated intravenous administration of RVG-9r-targeted SNALPs does not increase serum levels of IL-6. The levels of IL-6 for transgenic mice that were intravenously injected with a saline solution (HBS), RVG-9r-targeted liposomes encapsulating siCTR (siCTR) and RVG-9r-targeted liposomes encapsulating siMutAtax3 (siMutAtax3) were evaluated by ELISA. A wild-type animal injected with lipopolysaccharide (LPS) was used as a positive control. Values are presented as mean±SEM of *n*=4. One-way ANOVA analysis of variance combined with Bonferroni’s post-test was used to compare animals injected with HBS versus siMutAtax3 and siCTR (n.s. *p*>0.05).

**Animal**	**Transgenic**			**Wild Type**
**HBS-injected**	**siCTR**	**siMutAtax3**	**LPS**
**IL-6 levels (pg/mL)**	1.53±1.25	2.9±1.03	0.85±0.85	+500
